# A nitroreductase responsive probe for early diagnosis of pulmonary fibrosis disease

**DOI:** 10.1016/j.redox.2024.103294

**Published:** 2024-07-29

**Authors:** Shilan Peng, Yuanyuan Liang, Haotian Zhu, Yike Wang, Yun Li, Zuoquan Zhao, Yesen Li, Rongqiang Zhuang, Lumei Huang, Xianzhong Zhang, Zhide Guo

**Affiliations:** a State Key Laboratory of Vaccines for Infectious Diseases, Center for Molecular Imaging and Translational Medicine, Xiang an Biomedicine Laboratory, School of Public Health, Xiamen University, 4221-116 Xiang’An South Rd, Xiamen, 361102, China; b Theranostics and Translational Research Center, Institute of Clinical Medicine, Department of Nuclear Medicine, Peking Union Medical College Hospital, Chinese Academy of Medical Sciences & Peking Union Medical College, No. 1 Shuaifuyuan, Dongcheng District, Beijing, 100730, China; c Department of Nuclear Medicine & Minnan PET Center, The First Affiliated Hospital of Xiamen University, Xiamen, 361003, China

**Keywords:** Nitroreductase, Idiopathic pulmonary fibrosis, Molecular imaging, PET imaging, Reductive stress

## Abstract

Idiopathic pulmonary fibrosis (IPF) is a serious interstitial lung disease. However, the definitive diagnosis of IPF is impeded by the limited capabilities of current diagnostic methods, which may fail to capture the optimal timing for treatment. The main goal of this study is to determine the feasibility of a nitroreductase (NTR) responsive probe, ^18^F-NCRP, for early detection and deterioration monitoring of IPF. ^18^F-NCRP was obtained with high radiochemical purity (>95 %). BLM-injured mice were established by intratracheal instillation with bleomycin (BLM) and characterized through histological analysis. Longitudinal PET/CT imaging, biodistribution study and *in vitro* autoradiography were performed. The correlations between the uptake of ^18^F-NCRP and mean lung density (tested by CT), as well as histopathological characteristics were analyzed. In PET imaging study, ^18^F-NCRP exhibited promising efficacy in monitoring the progression of IPF, which was earlier than CT. The ratio of uptake in BLM-injured lung to control lung increased from 1.4-fold on D15 to 2.2-fold on D22. Biodistribution data showed a significant lung uptake of ^18^F-NCRP in BLM-injured mice. There was a strong positive correlation between the ^18^F-NCRP uptake in the BLM-injured lungs and the histopathological characteristics. Given that, ^18^F-NCRP PET imaging of NTR, a promising biomarker for investigating the underlying pathogenic mechanism of IPF, is attainable as well as desirable, which might lay the foundation for establishing an NTR-targeted imaging evaluation system of IPF.

## Graphical abstract

**Figure:**
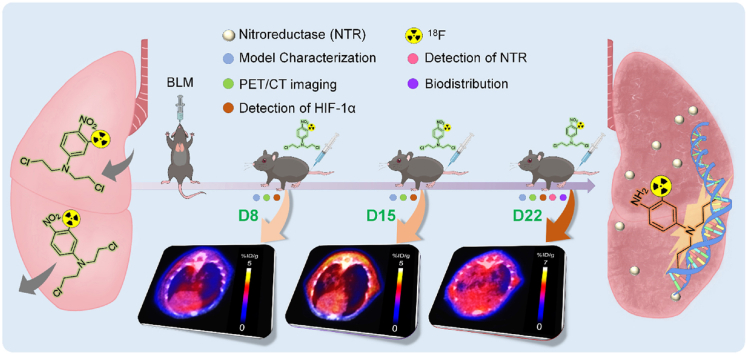

## 1 Introduction

Idiopathic pulmonary fibrosis (IPF) is classified as the most severe form of idiopathic interstitial pneumonias, associated with a median survival rate of 2.5–3.5 years [1]. Diagnosis as early as possible can allow patients to receive timely treatment and improve the decline in forced vital capacity [2,3]. High-resolution computed tomography (HRCT) plays a principal role in clinical setting to assess IPF, and histological examination can be omitted when the key CT features were found [4]. Regrettably, the features like honeycombing, reticular pattern, and traction bronchiectasis in lung images obtained by HRCT usually indicate an advanced stage of the disease [5,6]. According to clinical trials reported, there is a significant rate of missed diagnosis and misdiagnosis, with evidence suggesting it to be as high as 30%–50 % [7]. In cases where imaging results are atypical, lung biopsy is employed. However, some patients experience a poor prognosis, with high morbidity and mortality following the biopsy procedure [8]. Consequently, there is an urgent need to develop specific biomarkers and highly sensitive, non-invasive imaging techniques for early IPF diagnosis, disease progression monitoring, and prediction of disease behavior, which should help facilitate personalized treatment strategies for patients.

Positron emission tomography (PET) imaging is a real-time, noninvasive, and quantitative approach that has potential to accurately measure the severity of lung diseases and identify foci of metabolic activity, thereby improving diagnostic accuracy [[9], [10], [11]]. To date, as shown in Table 1, numerous radiotracers for IPF-targeted PET imaging have been reported, and several of them exhibit promising potential for diagnosis of IPF [[12], [13], [14], [15], [16], [17], [18], [19], [20], [21]]. The development of IPF-targeted radiotracers illustrates the necessity and urgency, and provides inspiration for the innovation of new biomarkers and probes.

**Table 1:** Overview of radiotracers for IPF PET imaging reported in the literatures.

Radiotracer	Target	Ref.
^18^F-FDG	Glucose transporters	[12]
^68^Ga-pentixafor	Chemokine receptor 4	[13]
^64^Cu-DOTA-ECL1i	Chemokine receptor 2	[14]
^18^F-FBEM	Sulfhydryl groups	[15]
^68^Ga-DOTA-NOC,^111^Ln-octreotide	Somatostatin receptors 2,3,5	[16,17]
^68^Ga-CBP8,^64^Cu-CBP1,3,5,6,7	Type 1 collagen	[18,19]
^18^F-FMISO	Hypoxic cells	[20]
^68^Ga-FAPI-46	Fibroblast activation protein	[21]

Recent evidences indicated the involvement of redox imbalance in the progression of IPF. In a state of health, cells diligently maintain a delicate balance between oxidants and antioxidants. Redox imbalance refers to the disruption of this dynamic equilibrium within the organism. Overproduction of oxidants leads to oxidative harm of cellular biomolecules, triggering oxidative stress, while insufficient oxidant levels disrupt essential signaling pathways, causing a state of reductive stress. This reductive stress can lead to an increase in reductase enzymes, such as nitroreductase (NTR) [22,23]. Several studies have shown that oxidative stress plays a crucial role in the pathogenesis of IPF [24]. This redox imbalance can activate the body's antioxidant system, and the stress-sensitive transcription factors such as nuclear factor erythroid 2-related factor (NRF2), nuclear factor kappa B (nf-kappa B) will be activated to produce NAD(P)H to reduce oxidative modification [25]. However, in the lungs of IPF, a hypoxic environment [26] inhibits the oxidation of NADH, leading to its accumulation and promoting the redox imbalance towards a state of reductive stress [27]. Furthermore, hypoxia can also lead to the occurrence of reductive stress by triggering glycogen synthesis in hypoxic tissue regions. Specifically, in hypoxic area, NADPH was produced through the pentose phosphate pathway (PPP), and the synthesis of reduced glutathione was promoted, which eventually leads to the occurrence of reducing stress [[28], [29], [30], [31]]. In addition, complete nitroreduction by oxygen-sensitive NTR must also occur under hypoxic conditions, and this conversion catalyzes the nitroreduction of nitroaromatic compounds by the addition of a single electron using NAD(P)H as an electron donor, producing transient nitroanion radicals, and ultimately ends up with a primary amine. Under hypoxia, the reduction process converts the electron-withdrawing nitro group into the electron-donating amino group, leading to a significant change in electron density. This change can serve as a selective "switch" mechanism to activate inert compounds. Consequently, the unique characteristics of NTR, namely demonstrating the existence of IPF involving stress reduction, make it a promising biomarker for the visualization of IPF.

Despite decades of studies on IPF, the exact pathogenesis mechanism remains elusive. Several previous reports have shown a possible association between redox imbalance and IPF, with special attention to the important role of reductive stress. These studies have sparked our interest in exploring the link between NTR, a target associated with reductive stress, and IPF, while developing visualization methods to assist in the diagnosis and treatment of IPF. To achieve the objectives, we established an IPF murine model induced by bleomycin (BLM), and then performed longitudinal PET/CT imaging to demonstrate the value of early diagnosis of IPF. We developed a nitrogen mustard analogue, the NTR responsive probe ^18^F-NCRP, as an imaging tool to assess disease activity. Moreover, this study conducted a series of *in vitro* and *in vivo* experiments to verify the correlation between NTR PET imaging and IPF disease progression, which might lay the foundation for the establishment of NTR-targeted imaging evaluation system for IPF.

## 2 Material and methods

### 2.1 General

All materials and reagents used in this study were purchased from commercial suppliers. [^18^F]fluoride ion was produced by cyclone cyclotron (IBA, Belgium) in the Center for Molecular Imaging and Translational Medicine of Xiamen University. The hydroxyproline (HYP) assay kit (Acmec) and picro-sirius red stain kit were purchased from Shanghai Acmec Biochemical Co., Ltd. DAPI (Beyotime, P0131) was purchased from Shanghai Beyotime Biotechnology Co., Ltd. HIF-1α (Santa Cruz, sc-13515) was purchased from Xiamen Xincheng Biotechnology Co., Ltd. High performance liquid chromatography (HPLC) analysis was performed on Dionex Ultimate 3000 HPLC (Thermo Scientific, USA) equipped with an Elysia Raytest Gabi Star γ-radiation detector. Imaging studies were performed using an Inveon microPET/CT scanner (Siemens Medical Solutions Inc., USA). Autoradiography images were acquired by a storage phosphor imager (Cyclone Plus, PerkinElmer Instruments Inc., USA). Biodistribution data were measured by γ-counter (PerkinElmer, USA). HYP result was determined using a microplate reader (Thermo Scientific, USA). Tissue immunofluorescence was performed by Zeiss LSM 880+Airyscan microscope (Carl Zeiss, Germany).

### 2.2 Chemistry and radiochemistry

^18^F-NCRP was synthesized according to previously published methods [32]. Briefly, 1.85 GBq [^18^F]fluoride ion was eluted from the preactivated QMA Carb cartridge (Waters light Sep-Pak, pretreated with 10 ml 0.5 M K_2_CO_3_ and 10 ml H_2_O) with 1 ml of eluate (900 μl CH_3_CN and 100 μl H_2_O containing 11 mg Kryptofix_222_ and 2 mg K_2_CO_3_) into the vial. Then, it was evaporated three times with anhydrous CH_3_CN under 110 °C to remove water. Next, 2 mg of the precursor dissolved in 1 ml anhydrous DMSO was added into the [^18^F]fluoride solution and then heated to 72 °C for 20 min. The crude product was diluted with 10 ml H_2_O and purified with a preactivated C18 (10 ml CH_3_OH and 10 ml H_2_O) and radio-HPLC (70 % CH_3_OH: 30 % H_2_O, 3 ml/min, UV = 254). ^18^F-NCRP was re-dissolved in saline with 5 % ethanol for further research. Radio-HPLC (70 % CH_3_OH: 30 % H_2_O, 1 ml/min, UV = 254) was used to determine the radiochemical purity (RCY).

### 2.3 NTR-specificity

The specificity of ^18^F-NCRP to NTR was determined through *in* *vitro* responsiveness assays using HPLC. The experiment was divided into four groups:

(I) 18.5 MBq of ^18^F-NCRP (dissolved in 100 μl of PBS), PBS (100 μl), catalysts NADPH (200 μM, 100 μl);
(Ⅱ) 9.25 MBq of ^18^F-NCRP, NTR (1.5 μg/ml, 100 μl), NADPH (200 μM, 100 μl);
(Ⅲ) 18.5 MBq of ^18^F-NCRP, NTR (1.5 μg/ml, 100 μl), NADPH (200 μM, 100 μl);
(Ⅳ) 18.5 MBq of ^18^F-NCRP, NTR (1.5 μg/ml, 100 μl), NADPH (200 μM, 100 μl), inhibitor dicumarol (0.59 mg/ml, 100 μl).

The mixture was incubated at 37.5 °C for 4 h under the atmosphere of nitrogen. Samples were analyzed by radio-HPLC (70 % CH_3_OH: 30 % H_2_O, 1 ml/min).

### 2.4 Animal model

All experimental procedures and the use of animals were carried out in compliance with the guidelines of the Animal Care and Use Committee of the Laboratory Animal Center of Xiamen University. BLM (Beyotime, Shanghai, China) was used to induce pulmonary fibrosis in mice. 10-week-old male C57BL/6J mice were given a single intratracheal injection of 2.5 mg/kg of BLM (dissolved in 50 μl 0.9 % normal saline, BLM-injured group) or 50 μl saline on day 0 (D0). PET imaging studies were conducted on day 8 (D8), day 15 (D15) and day 22 (D22).

### 2.5 Histochemical identification

**Hydroxyproline content:** A commercial kit was used to measure the levels of hydroxyproline content in the lung. In brief, lung tissues were digested with 6M HCl at 100 °C, followed by centrifugation, pH adjustment, volume determination, and color development. The microplate reader was adjusted to 560 nm for sample determination.

**Histological study:** Lung tissue specimens were fixed with 4 % paraformaldehyde overnight at 4–8 °C, then embedded with paraffin. The specimens were cut into 8-μm slices and then processed through xylene and graded ethanol (100 %, 95 %, 80 %, 75 % and 60 %) before being placed in double-distilled water. They were then stained with hematoxylin and eosin (H&E) or Sirius red, following the kit's instructions. Histologic images were acquired by the Leica DM4 B upright digital research microscopes (Leica).

**Immunofluorescence:** Lung tissues were frozen in optimal cutting temperature compound (O.C.T), stored at −80 °C. The specimens were cut into 8-μm slices by the freezing microtome at −20 °C. Tissue sections were fixed using 4 % paraformaldehyde for 10 min and washed three times in phosphate buffer saline (PBS). Nonspecific antibody binding was blocked by incubating the sections in 10 % goat serum for 30 min and washed in PBS three times. Subsequently, the tissue sections were incubated at 4 °C overnight with specific antibody of HIF-1α (mouse monoclonal, 1 : 50), and then incubated with Alexa fluor 488 anti-mouse IgG antibody as the secondary antibody (1 : 200) for 1 h. Cell nucleus was stained blue by antifade mounting medium (DAPI included). All the images were analyzed by ImageJ 7.0 software.

### 2.6 PET/CT imaging and autoradiography analysis

7.2 MBq of ^18^F-NCRP (200 μl) was intravenously injected into mice (n = 5). All the mice underwent 5-min static PET scans and 10-min micro-CT scans at 30 and 60 min post injection (p.i.). During the scanning, mice were subjected to gas anesthesia to maintain spontaneous breathing. To monitor the uptake in lung during various stage, longitudinal PET/CT imaging was performed weekly from D8 to D22. Three mice were selected on D22 and dicumarol (0.4 mM, 50 μl) was intratracheally instilled 1 h before ^18^F-NCRP injection to clear the NTR expression. The data were reconstructed using three-dimensional ordered-subset expectation-maximization (3D OSEM) algorithm. Regions of interest (ROI) were drawn on the decay-corrected whole body coronal images. All the mice were euthanized after PET/CT imaging on D22, and tissues were collected and exposed to obtain autoradiography images.

### 2.7 Biodistribution

Biodistribution of ^18^F-NCRP was performed on D22. Each mouse was injected with 0.7 MBq (100 μl) of ^18^F-NCRP via tail vein (n = 4). The BLM-injured mice were euthanized by decapitation at 30, 60, and 90 min p.i. Interested organs were collected, weighed, and radioactivity was counted using a γ-counter. The percentage of the injected dose per gram (%ID/g) of organs and tissues was calculated. The saline group served as the control.

### 2.8 Safety evaluation

The safety profile of ^18^F-NCRP was explored by animal experiments. Mice were randomly divided into four groups: 37 MBq of ^18^F-NCRP and same volume of saline for 3 male or 3 female mice per group, respectively. Weight was continuously monitored for 7 days. After that, the main organs of mice were collected for H&E staining to observe morphological changes, and the serum of each group was collected for blood tests to determine the liver and kidney function, including aspartate aminotransferase (AST), alanine aminotransferase (ALT), blood urea nitrogen (BUN) and creatinine (CR).

### 2.9 Statistical analysis

All data represent at least 4 independent mice, and quantitative data are expressed as the mean ± standard deviation (SD). Differences among groups were compared by a two-tailed Student's t-test. The Pearson correlation coefficient was computed between two quantitative variables (*p ≤ 0.05; **p ≤ 0.01; ***p ≤ 0.001; ****p ≤ 0.0001; ns, no significant difference).

## 3 Results

### 3.1 Chemistry and radiochemistry

**Scheme 1:**
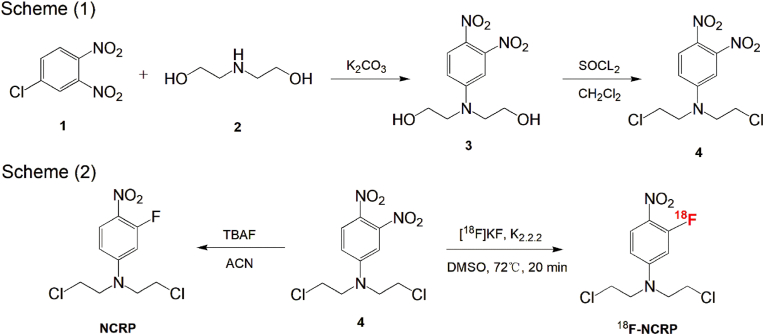
The synthetic routes of precursor, reference compound and ^18^F-NCRP.

The synthetic routes of precursor, reference compound, and ^18^F-NCRP are shown in Scheme 1. The compounds were confirmed by LC-MS and ^1^H NMR (Figs. S1–S3). The radiosynthesis of ^18^F-NCRP was completed within 50–60 min. After purification, ^18^F-NCRP and non-radioactive reference (^19^F-NCRP) were co-loaded into radio-HPLC. It was observed that their retention times were 9.56 and 9.31 min, respectively (Fig. S4). The RCP of ^18^F-NCRP after purification was >95 %. The radiochemical yield (RCY) without decay-corrected was 9.32 ± 1.87 %.

### 3.2 NTR-specificity

**Fig. 1:**
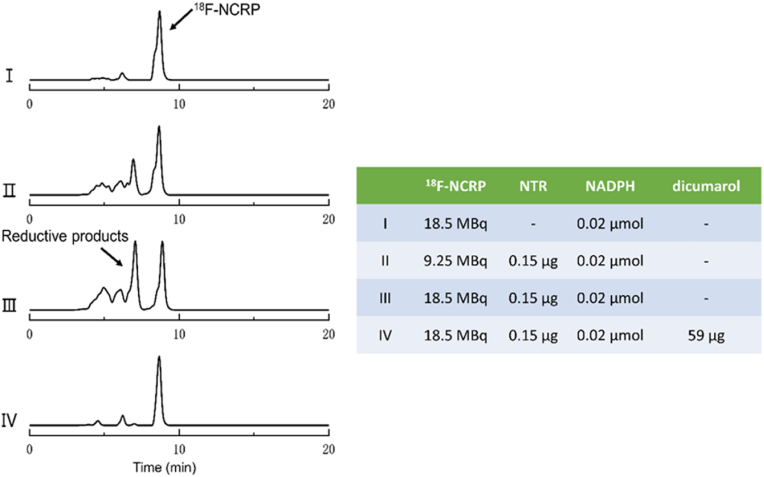
HPLC spectrum of the different reduction groups: (I) 18.5 MBq of ^18^F-NCRP (dissolved in 100 μl of PBS), PBS (100 μl), catalysts NADPH (200 μM, 100 μl); (II) 9.25 MBq of ^18^F-NCRP, NTR (1.5 μg/ml, 100 μl), NADPH (200 μM, 100 μl); (III) 18.5 MBq of ^18^F-NCRP, NTR (1.5 μg/ml, 100 μl), NADPH (200 μM, 100 μl); (IV) 18.5 MBq of ^18^F-NCRP, NTR (1.5 μg/ml, 100 μl), NADPH (200 μM, 100 μl), inhibitor dicumarol (0.59 mg/ml, 100 μl).

^18^F-NCRP was co-incubated with NTR in a hypoxic environment to verify responsiveness *in vitro*. In theory, the ^18^F-NCRP could react with NTR, reducing the proportion of the ^18^F-NCRP and simultaneously generating new reduction products. As expected, 68.96 % of ^18^F-NCRP was reduced in the presence of NTR and NADPH, while only 16.71 % reduction was achieved with the introduction of dicumarol. In addition, as the concentration of ^18^F-NCRP decreased, the fraction that underwent reduction also decreased. These results demonstrate that ^18^F-NCRP specifically respond to NTR *in vitro* (Fig. 1).

### 3.3 Characterization of BLM-injured mice

After a single intratracheal injection with BLM, the lungs of mice exhibited significant damage. H&E staining (Fig. S5A) revealed a marked disappearance of alveolar structure and a noticeable increase in fibroblast proliferation and extracellular matrix over time in the fibrosis group (D8 - D22). In contrast, the saline group showed no apparent lesions. Sirius red staining (Fig. S5B) demonstrated prominent red-colored collagen deposition with a widespread distribution in the lungs of BLM-injured mice following the inflammatory phase. The quantification data showed a significant difference between the two groups of mice at D8 (saline: 2.42 ± 0.71 %, BLM: 9.53 ± 4.84 %; *p* < 0.001), D15 (saline: 2.16 ± 1.40 %, BLM: 16.32 ± 7.08 %; *p* < 0.0001), and D22 (saline: 3.25 ± 2.97 %, BLM: 19.11 ± 3.92 %; *p* < 0.0001) (Fig. S5C). Lung tissues in BLM-injured mice were also dissected to measure HYP content. A significant increase was observed at D8 (16.56 ± 1.31 mg/kg), D15 (18.88 ± 0.72 mg/kg), and D22 (21.34 ± 1.43 mg/kg), compared to the control (Fig. S5D). The Ashcroft histopathological score exhibited a similar trend (Fig. S5E). Compared to the control, the BLM-injured lung showed a significantly higher degree of fibrosis (*p* < 0.0001).

CT images of BLM-injured mice exhibited significant bilateral asymmetric ground-glass opacity in the lung, which progressively diffused over time, contrasting with the clear lung field in both sides of control (Fig. S6A). This indicated a heightened severity of pulmonary fibrosis. Substantial differences in mean lung density between the two groups were observed on D8 (saline: -408.24 ± 12.58 HU, BLM: -387.98 ± 13.05 HU; *p* < 0.01), D15 (saline: -383.67 ± 16.56 HU, BLM: -341.68 ± 13.12 HU; *p* < 0.0001) and D22 (saline: -407.78 ± 7.89 HU, BLM: -309.09 ± 26.12 HU; *p* < 0.0001) (Fig. S6B). Additionally, the results indicated a moderate correlation between mean lung density and the Sirius red positive area value (r = 0.65, *p* < 0.001) (Fig. S6C), as well as the HYP quantitative value (r = 0.78, *p* < 0.001) (Fig. S6D).

### 3.4 PET/CT imaging

**Fig. 2:**
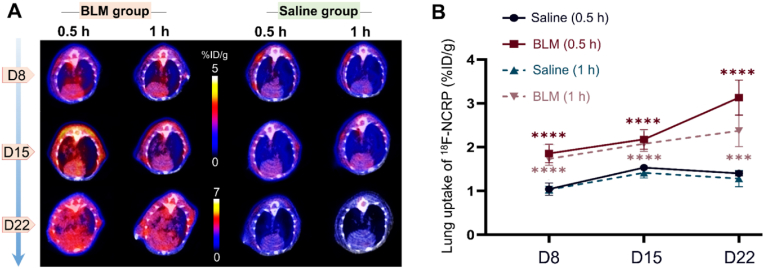
PET/CT imaging of ^18^F-NCRP in BLM-injured and saline group. (A) Representative PET/CT images (horizontal view) of ^18^F-NCRP in BLM-injured and saline mice (n = 4). (B) Quantification of ^18^F-NCRP uptake in the lung tissues. The difference between the BLM-injured group and the saline group at 0.5 h (bold) and 1 h (light) was compared. ***p < 0.001, ****p < 0.0001.

To assess the potential of ^18^F-NCRP for early monitoring of IPF disease progression, PET/CT imaging studies were conducted on D8, D15, and D22. Significant lung uptake of ^18^F-NCRP in BLM-injured mice was showed in Fig. 2A. The radioactivity in lung increased in correlation with the escalating severity of pulmonary fibrosis observed on CT images. Specifically, the BLM-injured lung uptake of ^18^F-NCRP on D8 was 1.86 ± 0.21%ID/g at 0.5 h p.i., which was slightly higher than that in the control group (1.04 ± 0.14%ID/g, *p* < 0.0001). Moreover, the disparity between the two groups significantly increased to 2.2 times on D22 (3.13 ± 0.40 *vs* 1.40 ± 0.05%ID/g, *p* < 0.0001). Meanwhile, the lung uptake in the dicoumarol-treated mice at 0.5 h p.i. was only 1.64 ± 0.14%ID/g (Fig. S7). Compared to 0.5 h p.i., the lung uptake slightly declined in both groups at 1 h p.i. (D8, 1.73 ± 0.15 *vs* 1.03 ± 0.08%ID/g, *p* < 0.0001; D15, 2.08 ± 0.17 *vs* 1.42 ± 0.12%ID/g, *p* < 0.0001; D22, 2.38 ± 0.36 *vs* 1.28 ± 0.19%ID/g, *p* < 0.001) (Fig. 2B).

### 3.5 Autoradiography analysis

**Fig. 3:**
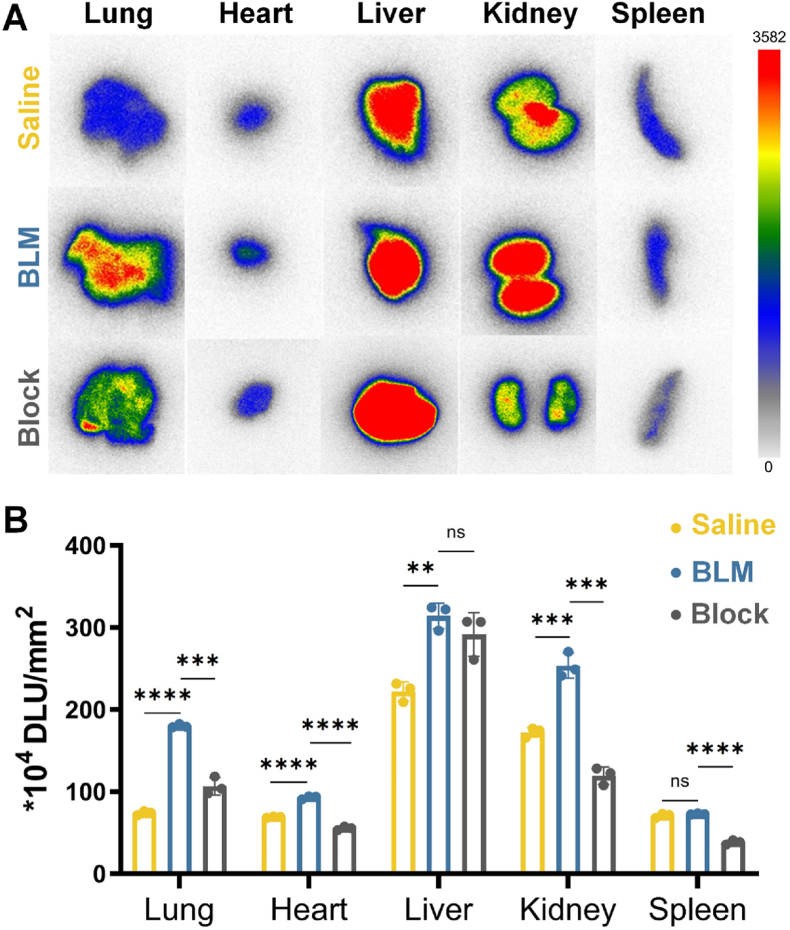
(A) Autoradiography of interest organs in saline, BLM-injured and blocking groups after injected with ^18^F-NCRP. (B) Quantification of *ex vivo* autoradiography for ^18^F-NCRP in the interest organs of different groups on D22. *p < 0.05, **p < 0.01, ***p < 0.001, ****p < 0.0001; ns, no significant difference.

As depicted in Fig. 3A, to further study the distribution of the radiotracer, lung and some other organs of interest were collected for autoradiography analysis after PET/CT imaging on D22. A significant uptake was observed in BLM-injured lung ((180.09 ± 2.22) × 10^4^ DLU/mm^2^), which was 2.47-fold higher than in the saline group ((73.85 ± 2.62) × 10^4^ DLU/mm^2^). Lung uptake was significantly decreased in blocked group ((106.56 ± 8.68) × 10^4^ DLU/mm^2^; *p* < 0.001). Additionally, statistically differences were also observed in other organs, such as the heart (BLM: (92.77 ± 2.06) × 10^4^ DLU/mm^2^ *vs* saline: (69.10 ± 0.76) × 10^4^ DLU/mm^2^; *p* < 0.0001), liver (BLM: (314.28 ± 15.63) × 10^4^ DLU/mm^2^ *vs* saline: (222.20 ± 11.68) × 10^4^ DLU/mm^2^; *p* < 0.01), and kidney (BLM: (253.50 ± 15.24) × 10^4^ DLU/mm^2^ *vs* saline: (172.26 ± 5.81) × 10^4^ DLU/mm^2^; *p* < 0.001) (Fig. 3B).

### 3.6 Biodistribution

**Fig. 4:**
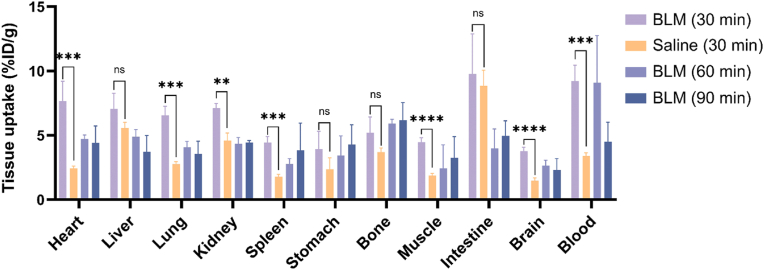
Biodistribution of ^18^F-NCRP in BLM and saline-treated mice on D22 (n = 4). **p < 0.01, ***p < 0.001, ****p < 0.0001; ns, no significant difference.

The biodistribution data of ^18^F-NCRP in both BLM and saline-treated mice are shown in Fig. 4 and Table S1. At 30 min p.i., obvious radioactivity uptake was observed in BLM-injured lung, which was significantly higher than that of saline group (6.55 ± 0.70 *vs* 2.76 ± 0.17%ID/g, *p* < 0.001). The radioactivity in lung slightly decreased at 60 min p.i. (4.07 ± 0.46%ID/g) and further decreased at 90 min p.i. (3.56 ± 0.99%ID/g). Higher uptakes of radiotracer in BLM-injured mice organs were also observed at 30 min p.i., such as blood (9.21 ± 1.24 *vs* 3.39 ± 0.22%ID/g, *p* < 0.001), heart (7.67 ± 1.54 *vs* 2.43 ± 0.15%ID/g, *p* < 0.001), kidneys (7.11 ± 0.37 *vs* 4.60 ± 0.51%ID/g, *p* < 0.01), spleen (4.43 ± 0.49 *vs* 1.79 ± 0.16%ID/g, *p* < 0.001), muscle (4.47 ± 0.35 *vs* 1.88 ± 0.14%ID/g, *p* < 0.0001) and brain (3.77 ± 0.30 *vs* 1.48 ± 0.19%ID/g, *p* < 0.0001). The lung/other organ ratios were calculated, and multiple t-tests between the two groups revealed statistical differences with lung/liver, lung/kidney and lung/heart (Fig. S8).

### 3.7 Hypoxia analysis of pulmonary fibrosis

**Fig. 5:**
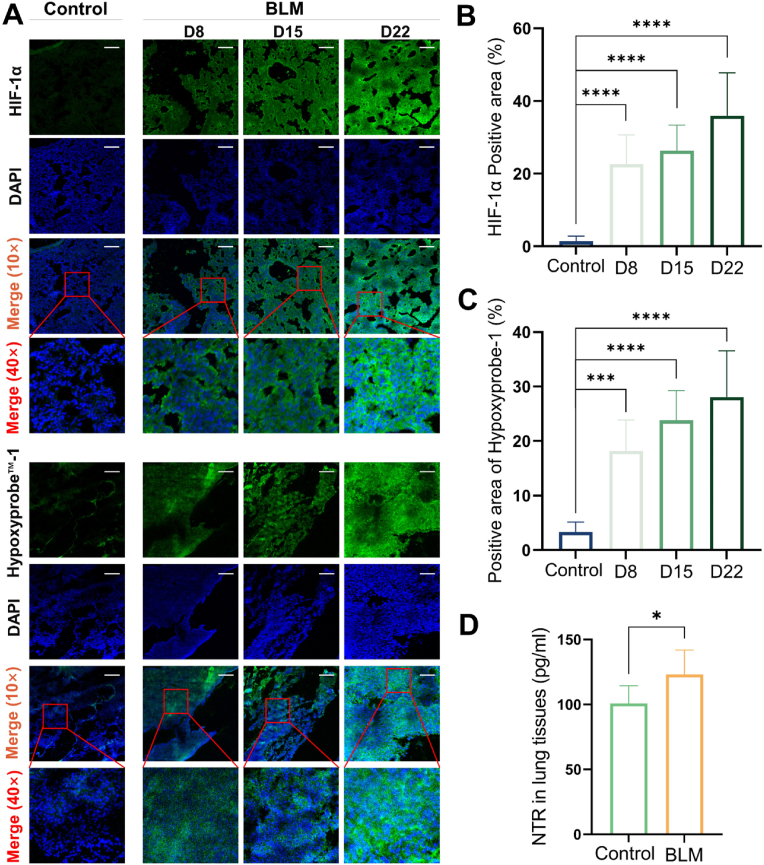
Detection of hypoxic signal and NTR expression. (A) Representative immunofluorescent images of hypoxia (above) and Hypoxyprobe™-1 (below) analysis in lung tissues. HIF-1α and Hypoxyprobe™-1 (green), DAPI (blue). Scale bar: 100 μm. (B, C) Corresponding quantitative data for HIF-1α and Hypoxyprobe™-1 on D8, D15 and D22. (D) ELISA of NTR in lung tissues on D22. *p < 0.05, ***p < 0.001, ****p < 0.0001. (For interpretation of the references to color in this figure legend, the reader is referred to the Web version of this article.)

Immunofluorescence staining of BLM-injured lung was conducted. As showed in Fig. 5A, the fluorescence signal of HIF-1α in the lungs of BLM-injured mice was significantly enhanced as pulmonary fibrosis worsened. The expression levels of HIF-1α were measured by the positive area (D8: 22.63 ± 8.06 %, D15: 26.33 ± 7.04 %, D22: 35.90 ± 11.86 %). However, the signal of control was weaker (1.42 ± 1.41 %, *p* < 0.0001) (Fig. 5B). Hypoxyprobe™-1 (Pimonidazole) was employed to detect NTR in lung tissues, which is a technique that exploits the ability of Pimonidazole to selectively bind to NTR in the area of hypoxic cells for measuring hypoxia area at the cellular level. Results showed that the expression of NTR in control group (3.32 ± 1.63 %) was lower than in that of BLM-injured mice (D8: 18.20 ± 4.91 %, *p* < 0.001; D15: 23.82 ± 4.95 %, *p* < 0.0001; D22: 28.08 ± 7.91 %, *p* < 0.0001) (Fig. 5A and **C**). Concordant results were observed in ELISA analysis. Fig. 5D showed that the NTR level in the lungs of BLM-injured mice on D22 (123.10 ± 18.72 pg/ml) was higher than that in saline-treated mice (100.68 ± 13.67 pg/ml; *p* < 0.05).

### 3.8 Correlation analysis

**Fig. 6:**
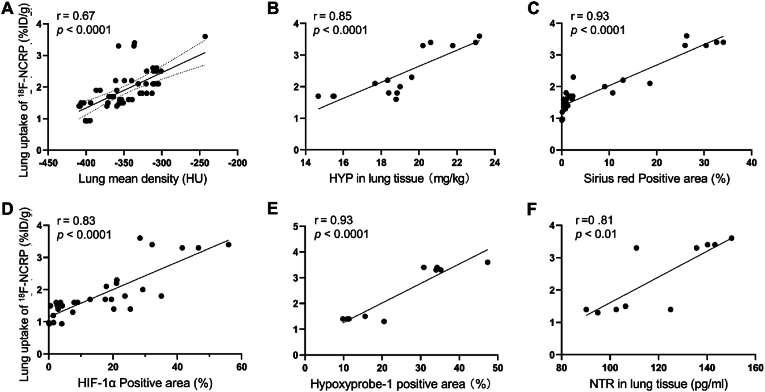
Correlation analysis of PET imaging and histopathological characteristics. Correlation analysis between BLM-injured lung uptake of ^18^F-NCRP and lung mean density (A), HYP content (B), Sirius red positive area (C), HIF-1α positive area (D), hypoxyprobe™-1 positive area (E), NTR content (F).

Based on the above results, we did a correlation analysis between the BLM-injured lung uptake of ^18^F-NCRP and lung mean density, HYP content, Sirius red positive area, HIF-1α positive area, Hypoxyprobe™-1 positive area and NTR content. As expected, a good correlation was observed between the ^18^F-NCRP uptake of BLM-injured lung and CT data (r = 0.67, *p* < 0.0001) (Fig. 6A). Similarly, significant correlations were revealed between the radioactivity uptake and the HYP content (r = 0.85, *p* < 0.0001), and the quantitative data of Sirius red (r = 0.93, *p* < 0.0001) (Fig. 6B and **C**). Furthermore, results indicated a noteworthy positive association between the lung uptake of ^18^F-NCRP and the expression levels of HIF-1α (r = 0.83, *p* < 0.0001), Hypoxyprobe-1 (r = 0.93, *p* < 0.0001), and NTR (r = 0.81, *p* < 0.01) (Fig. 6D–**6F**).

### 3.9 Safety of ^18^F-NCRP

The curves of body weight changes in different groups of mice after administration of ^18^F-NCRP (37 MBq) are shown in Fig. S9. Both the female and male mice injected with saline or ^18^F-NCRP showed similar trends in body weight changes and all mice survived throughout the seven-day observation period. In addition, the results of blood routine tests showed that the indexes of liver and kidney function in all mice were within the normal range (Tables S2–S5), indicating that ^18^F-NCRP did not cause damage to liver and kidney function. H&E staining of major organs showed no organic lesions in each group of mice (Fig. S10), indicating the lack of evident toxicological effects associated with ^18^F-NCRP.

## 4 Discussion

The exact pathogenic mechanism of IPF remains elusive, with academic opinions diverging due to the complexity of signaling pathways and involvement of numerous cell types [33,34]. As the most prevalent form of interstitial lung disease, IPF exhibits an incidence rate ranging from 4.6 to 17 cases per 100,000 people per year [35,36]. Acute exacerbations occur in approximately 10 %–20 % of patients annually [37]. The appearance of honeycombing, a key diagnostic feature on HRCT, typically signifies the end-stage of lung fibrosis. However, HRCT can be challenging when patients also have concurrent emphysema, potentially leading to delays in treatment initiation [38]. Therefore, there is an urgent need to develop more sensitive and non-invasive approaches for the early detection of IPF. Furthermore, the identification of a sensitive and specific biomarker to characterize the disease endotype is of paramount importance.

In this study, the BLM-injured mice were characterized through histological analysis (Fig. 1). Consistent with previous research, we observed destruction of the alveolar structure and deposition of extracellular matrix, accompanied by a rise of HYP content from D8 to D22 [39]. Compared with saline group, conspicuous red collagen fiber bundles were evident in the lung of BLM-injured mice. While much research on IPF has centered on redox imbalance, particularly focusing on signaling pathways involving NADPH oxidases, eosinophil peroxidase, mitochondrial electron transport chain, and myeloperoxidase, which can be activated to generate ROS [40,41], our study based on the expression of hypoxia signal in the IPF model, boldly demonstrated that the dynamic changes of redox imbalance, especially reductive stress, during the evolution of IPF disease could be monitored by PET imaging. HIF-1α emerges as the primary factor in the context of hypoxia, and our findings indicate that its expression increases with the severity of fibrosis, underscoring the presence of a hypoxic environment in the fibrotic lung. This observation is consistent with the research conducted by Tzouvelekis A et al. [42]. Furthermore, we validated our hypothesis using ^18^F-NCRP, which has been shown to specifically respond to NTR in this study. Our findings underscore the expression of NTR in the BLM-induced lung tissues (Fig. 5), which positively correlated with the lung uptake of ^18^F-NCRP (Fig. 6).

**Fig. 7:**
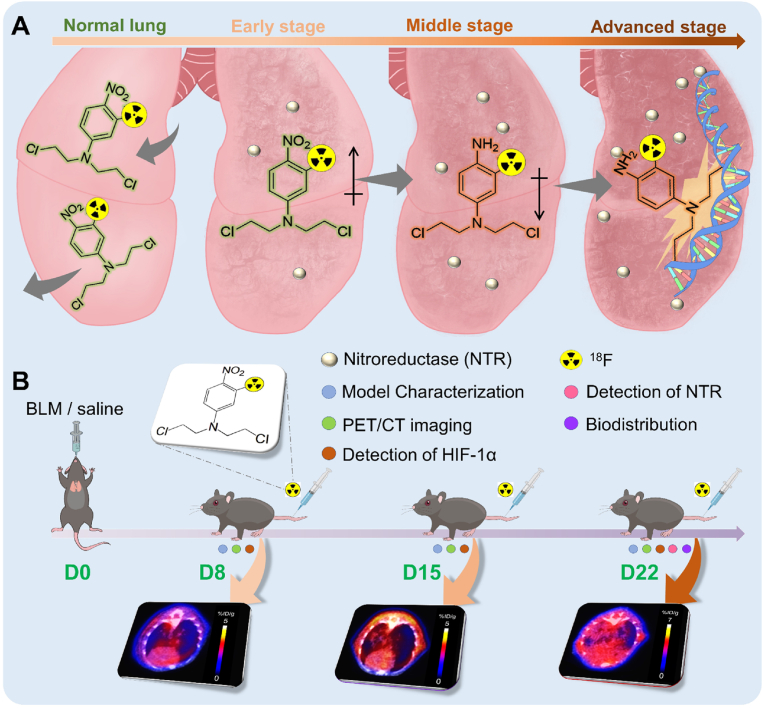
(A) Proposed mechanism of NTR-activated ^18^F-NCRP in BLM-injured lung at various stages. (B) Schematics of the procedures and timelines of PET imaging for BLM-injured mice. Saline group served as the control.

As illustrated in Fig. 7, the primary objective of this study was to assess the potential of ^18^F-NCRP for early detection and dynamic monitoring of the IPF progression. In NTR-rich lesions of lung fibrosis, the phenyl nitro group on ^18^F-NCRP undergoes specific reduction to an electron-donating amino group, activating the nitrogen mustard group to cross-link with DNA, leading to the retention of radioactivity in the IPF region. Conversely, the selective “switch” mechanism remains "OFF" in normal organs. Consequently, PET imaging with ^18^F-NCRP demonstrated promising results in the early detection of IPF. As shown in Fig. 2 and Fig. S6, PET imaging with ^18^F-NCRP can detect IPF lesions earlier than CT, potentially extending the time window and allow for the implementation of more assertive therapeutic interventions. Moreover, the ratio of uptake in BLM-injured lung to control lung increased from 1.4-fold on D15 to 2.2-fold on D22, indicating that the ^18^F-NCRP PET allows for continuous monitoring of IPF advancement. Importantly, a strong positive correlation was observed between the uptake of ^18^F-NCRP in BLM-injured lungs and histopathological characteristics. This correlation underscores the potential utility of ^18^F-NCRP PET imaging in evaluating the pathogenesis of IPF.

The *ex vivo* findings corroborated the imaging outcomes. In biodistribution (Fig. 4 and Table S1), the uptake of ^18^F-NCRP in BLM-injured lung was 2.37 times higher than that in the control group at 30 min p.i. Notably, compared to the saline-treated group, there was a significant increase in the uptake of ^18^F-NCRP in the heart and blood. One plausible explanation for this observation could be the development of chronic hypoxic pulmonary hypertension as a complication in the BLM-injured model [43,44]. Chronic hypoxia-induced pulmonary vasoconstriction is a known mechanism underlying pulmonary hypertension development [45,46]. Therefore, it is reasonable to infer an association between NTR expression and the manifestation of reductive stress in chronic hypoxic pulmonary hypertension. Consequently, ^18^F-NCRP selectively accumulates in the hypoxic area where NTR is expressed. These findings underscore the specificity of ^18^F-NCRP as a promising tool for the diagnosis and monitoring the progression of IPF, thus opening new avenues for exploring the pathogenic mechanism of IPF through the study of reductive stress. Moreover, it suggests the feasibility of targeting NTR for IPF PET imaging.

^18^F-NCRP, functioning as a radiotracer, offers real-time and noninvasive visualization through PET, enabling the detection of metabolic activity in lung foci in a gentle and prompt manner. However, a limitation of this study is that the fibrosis induced by BLM may not fully replicate the irreversible and complex course observed in IPF patients, and the underlying pathogenesis may differ slightly. In future investigations, more appropriate modeling methods will be explored to better understand the mechanisms of IPF in animal models. Concurrently, various animal modeling techniques will be explored to validate the efficacy of ^18^F-NCRP. Furthermore, forthcoming research efforts will delve into exploring the correlation between ^18^F-NCRP PET imaging and the therapeutic responses to drugs like nintedanib or pirfenidone, aiming to provide more precise guidance for personalized treatment [47].

In summary, ^18^F-NCRP demonstrates promising performance in IPF detection. PET imaging reveals a notable lung uptake of ^18^F-NCRP during the progressive stage of IPF. Compared to the traditional CT imaging, ^18^F-NCRP PET imaging can detect IPF at an earlier stage. Moreover, the uptake of ^18^F-NCRP in BLM-injured lungs exhibits a positive correlation with histopathological characteristics. Hence, ^18^F-NCRP PET imaging of NTR, a promising biomarker for investigating the underlying pathogenic mechanism of IPF, is both achievable and desirable.

## CRediT authorship contribution statement

**Shilan Peng:** Writing – original draft, Visualization, Software, Project administration, Methodology, Formal analysis, Data curation, Conceptualization. **Yuanyuan Liang:** Writing – original draft, Visualization, Software, Project administration, Methodology, Formal analysis, Data curation, Conceptualization. **Haotian Zhu:** Project administration, Data curation. **Yike Wang:** Project administration, Data curation. **Yun Li:** Project administration, Data curation. **Zuoquan Zhao:** Writing – review & editing. **Yesen Li:** Writing – review & editing. **Rongqiang Zhuang:** Writing – review & editing. **Lumei Huang:** Writing – review & editing, Visualization, Supervision, Resources, Project administration, Methodology, Funding acquisition, Formal analysis, Data curation, Conceptualization. **Xianzhong Zhang:** Writing – review & editing, Visualization, Supervision, Resources, Project administration, Methodology, Funding acquisition, Formal analysis, Data curation, Conceptualization. **Zhide Guo:** Writing – review & editing, Visualization, Supervision, Resources, Project administration, Methodology, Funding acquisition, Formal analysis, Data curation, Conceptualization.

## Declaration of competing interest

The authors declare that they have no conflict of interest.

## Data Availability

Data will be made available on request.

## Appendix A Supplementary data

Additional experimental details, including the HPLC, MS and ^1^H NMR spectrum; NTR-specificity; CT imaging of lung tissues in IPF and saline mice; biodistribution results of ^18^F-NCRP in BLM-injured and saline groups; safety data of ^18^F-NCRP.
