# Supplementary material for: A nitroreductase responsive probe for early diagnosis of pulmonary fibrosis disease
Source: Redox Biol. 2024 Jul 29;75:103294. doi: 10.1016/j.redox.2024.103294 (PMC11345524; doi:10.1016/j.redox.2024.103294)
Supplement: Multimedia component 1 [file mmc1.docx]

***Supporting information***

**A** **Nitroreductase Responsive Probe for Early Diagnosis of** **Pulmonary Fibrosis Disease**

Shilan Peng,^1,§^ Yuanyuan Liang,^1,§^ Haotian Zhu,^1^ Yike Wang,^1^ Yun Li,^1^ Zuoquan Zhao,^2^ Yesen Li,^3^ Rongqiang Zhuang,^1^ Lumei Huang,^1,^* Xianzhong Zhang,^2,^* and Zhide Guo^1,^*

1. State Key Laboratory of Vaccines for Infectious Diseases, Center for Molecular Imaging and Translational Medicine, Xiang An Biomedicine Laboratory, School of Public Health, Xiamen University, 4221-116 Xiang’An South Rd, Xiamen 361102, China.

2. Theranostics and Translational Research Center, Institute of Clinical Medicine, Department of Nuclear Medicine, Peking Union Medical College Hospital, Chinese Academy of Medical Sciences & Peking Union Medical College, No. 1 Shuaifuyuan, Dongcheng District, Beijing 100730, China.

3. Department of Nuclear Medicine & Minnan PET Center, The First Affiliated Hospital of Xiamen University, Xiamen 361003, China.

* **Corresponding authors:**

Zhide Guo, Email: gzd666888@xmu.edu.cn

Xianzhong Zhang, E-mail: zhangxzh@pumch.cn

Lumei Huang, Email: huanglmchemistry@126.com

**Table of Contents**

| **Contents:** | **Page(s):** |
| --- | --- |
| **Figure S1.** The MS of compound 1. | P. S3 |
| **Figure S2.** The ^1^H NMR spectrum of the compound 1. | P. S4 |
| **Figure S3.** The ^1^H NMR spectrum of the NCRP. | P. S5 |
| **Figure S4.** HPLC spectrum of the mixture of ^18^F-NCRP with the non-radioactive reference ^19^F-NCRP | P. S6 |
| **Figure S5**. HPLC spectrum of the different reduction groups | P. S7 |
| **Figure S6.** Longitudinal CT imaging of lung tissues in IPF and saline mice. | P. S8 |
| **Figure S7.** PET/CT imaging of ^18^F-NCRP in BLM-injured mice with NTR cleared. | P. S9 |
| **Figure S8.** Comparison of lung-to-tissue ratios from biodistribution of BLM-injured and saline groups at 30 min on D22. | P. S10 |
| **Figure S9**. Weight change curve of mice after injection of 37 MBq of ^18^F-NCRP. | P. S11 |
| **Figure S10.** H&E staining of major organs in different group on the D7 after injection of ^18^F-NCRP (37 MBq). | P. S12 |
| **Table S1.** Biodistribution results of ^18^F-NCRP in BLM-injured and saline groups. | P. S13 |
| **Table S2**. ALT activity of mice serum was detected on the D7 after injection 37 MBq of ^18^F-NCRP. | P. S14 |
| **Table S3**. AST activity of mice serum was detected on the D7 after injection 37 MBq of ^18^F-NCRP. | P. S15 |
| **Table S4**. BUN activity of mice serum was detected on the D7 after injection 37 MBq of ^18^F-NCRP. | P. S16 |
| **Table S5**. CR activity of mice serum was detected on the D7 after injection 37 MBq of ^18^F-NCRP. | P. S17 |


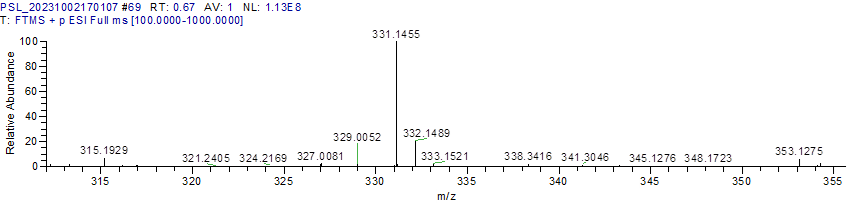


**Figure S1**. The MS of compound 1.


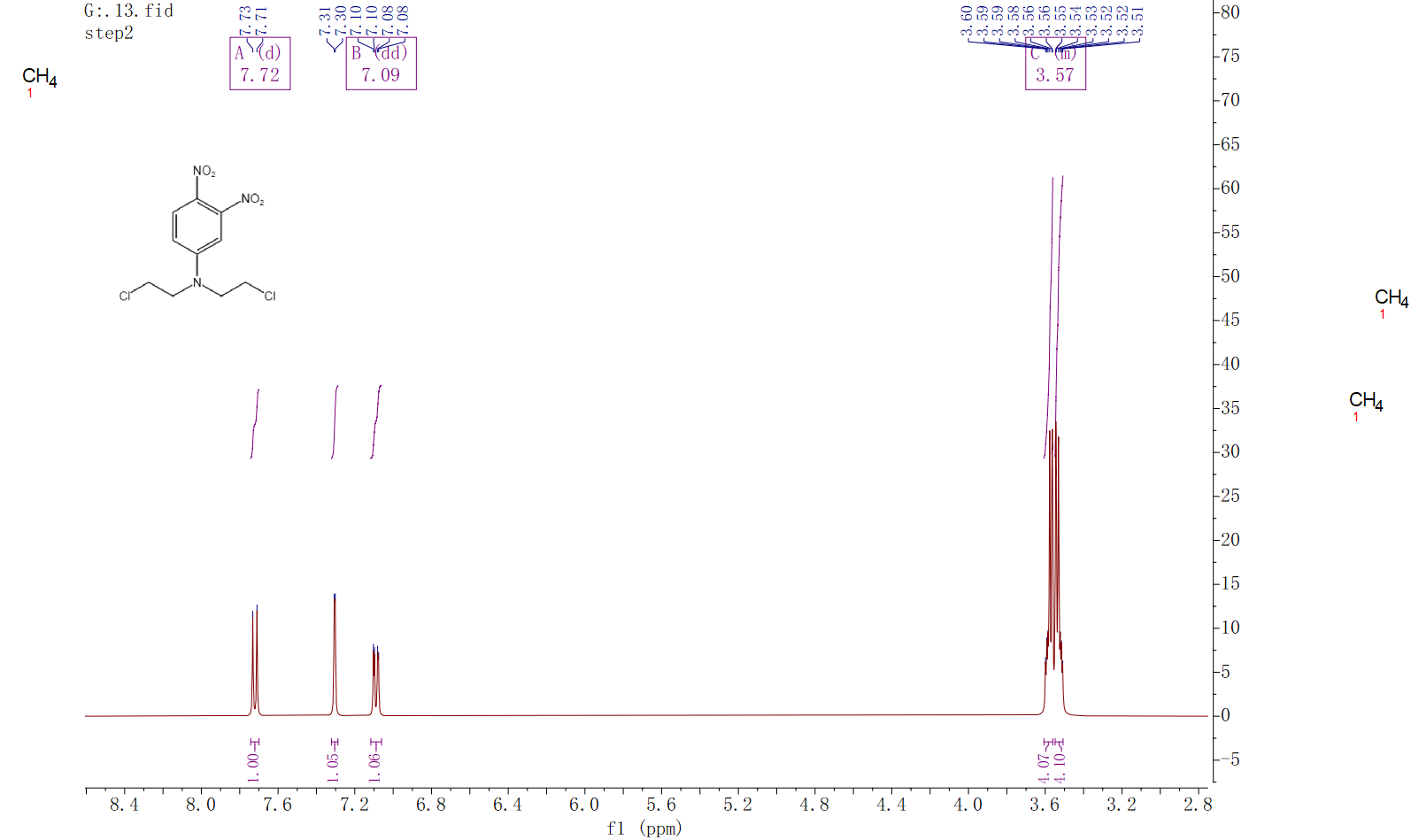


**Figure S2**. The ^1^H NMR spectrum of the compound 1. ^1^H NMR (400 MHz, CDCl_3_) δ 7.72 (d, *J* = 8.7 Hz, 1H), 7.30 (d, *J* = 2.1 Hz, 1H), 7.09 (dd, *J* = 8.7, 2.1 Hz, 1H), 3.60 – 3.56 (m, 4H), 3.55 – 3.51 (m, 4H).


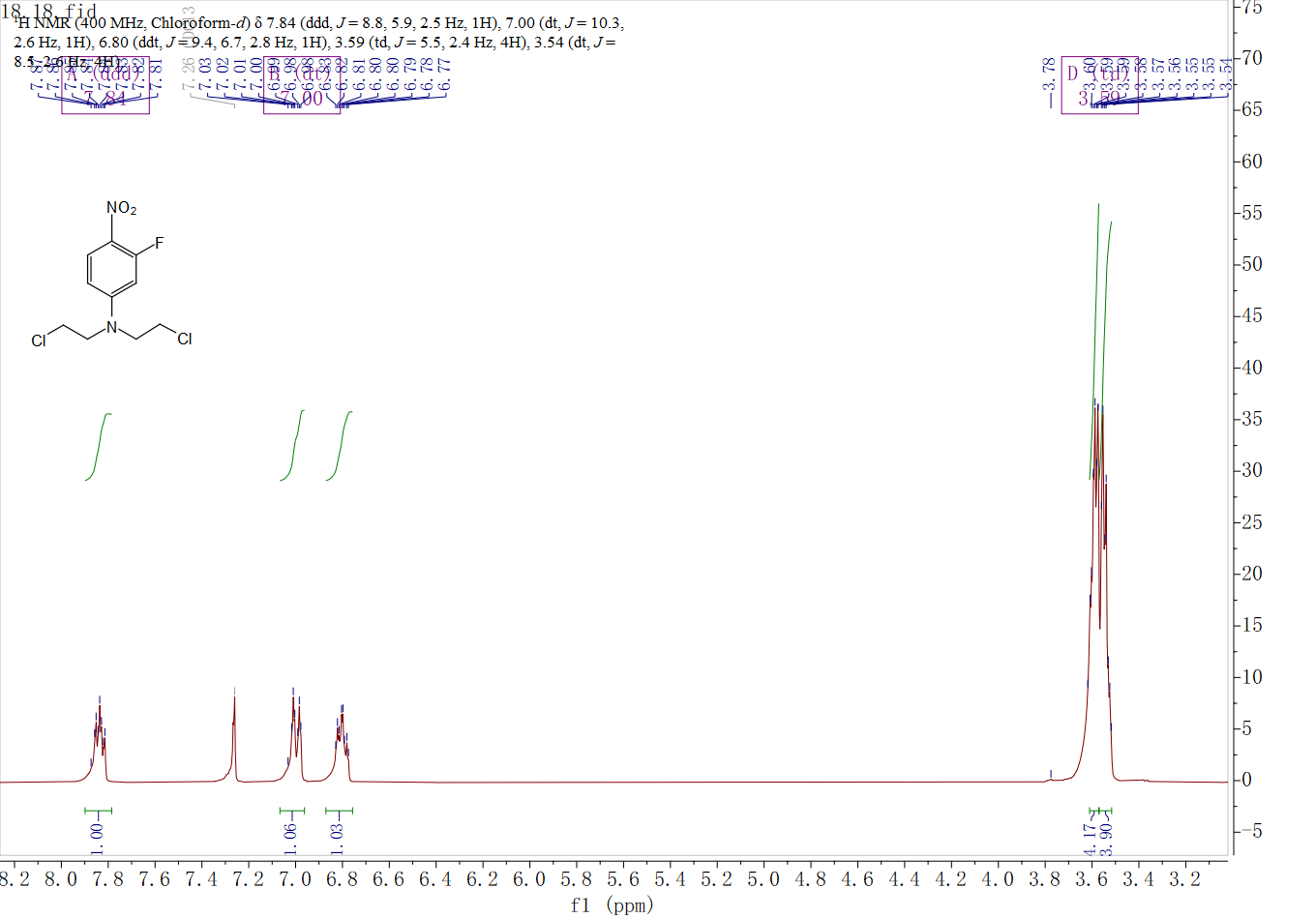


**Figure S3**. The ^1^H NMR spectrum of the NCRP. ^1^H NMR (400 MHz, CDCl_3_) δ 7.84 (ddd, J = 8.8, 5.9, 2.5 Hz, 1H), 7.00 (dt, *J* = 10.3, 2.6 Hz, 1H), 6.80 (ddt, *J* = 9.4, 6.7, 2.8 Hz, 1H), 3.59 (td, *J* = 5.5, 2.4 Hz, 4H), 3.54 (dt, *J* = 8.5, 2.6 Hz, 4H).


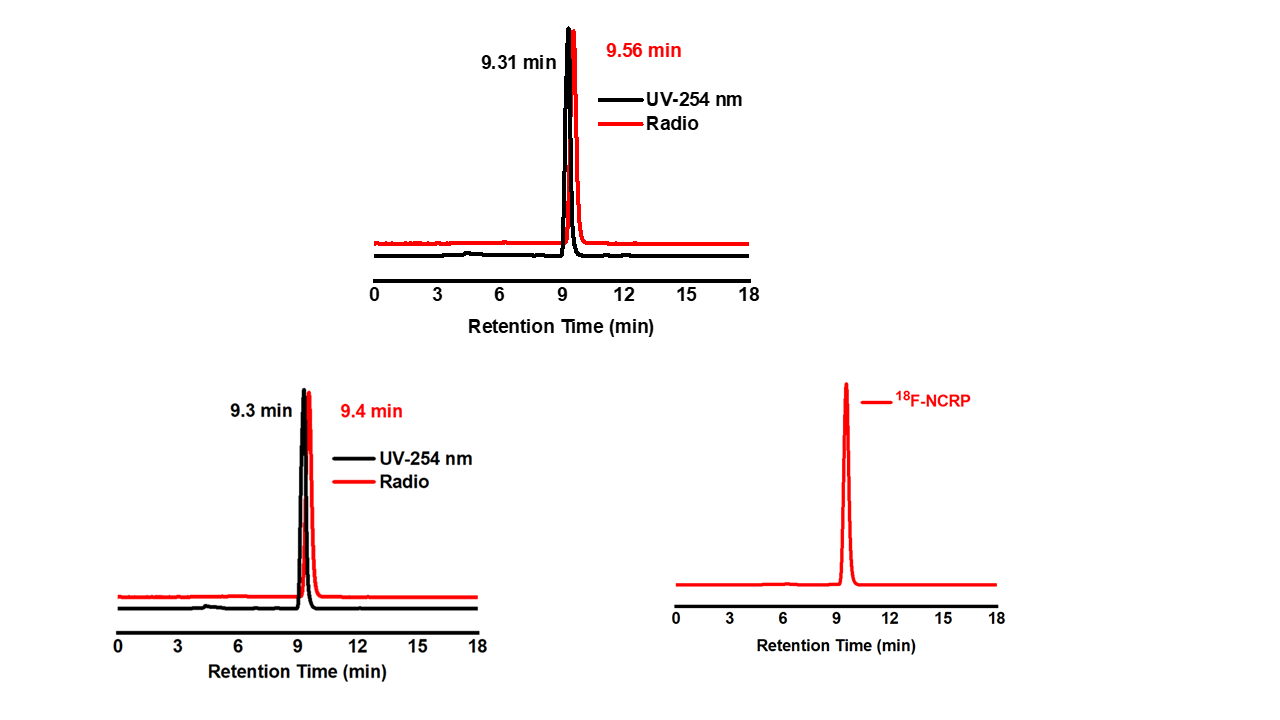


**Figure S4**. HPLC spectrum of the mixture of ^18^F-NCRP with the non-radioactive reference ^19^F-NCRP (UV = 254).


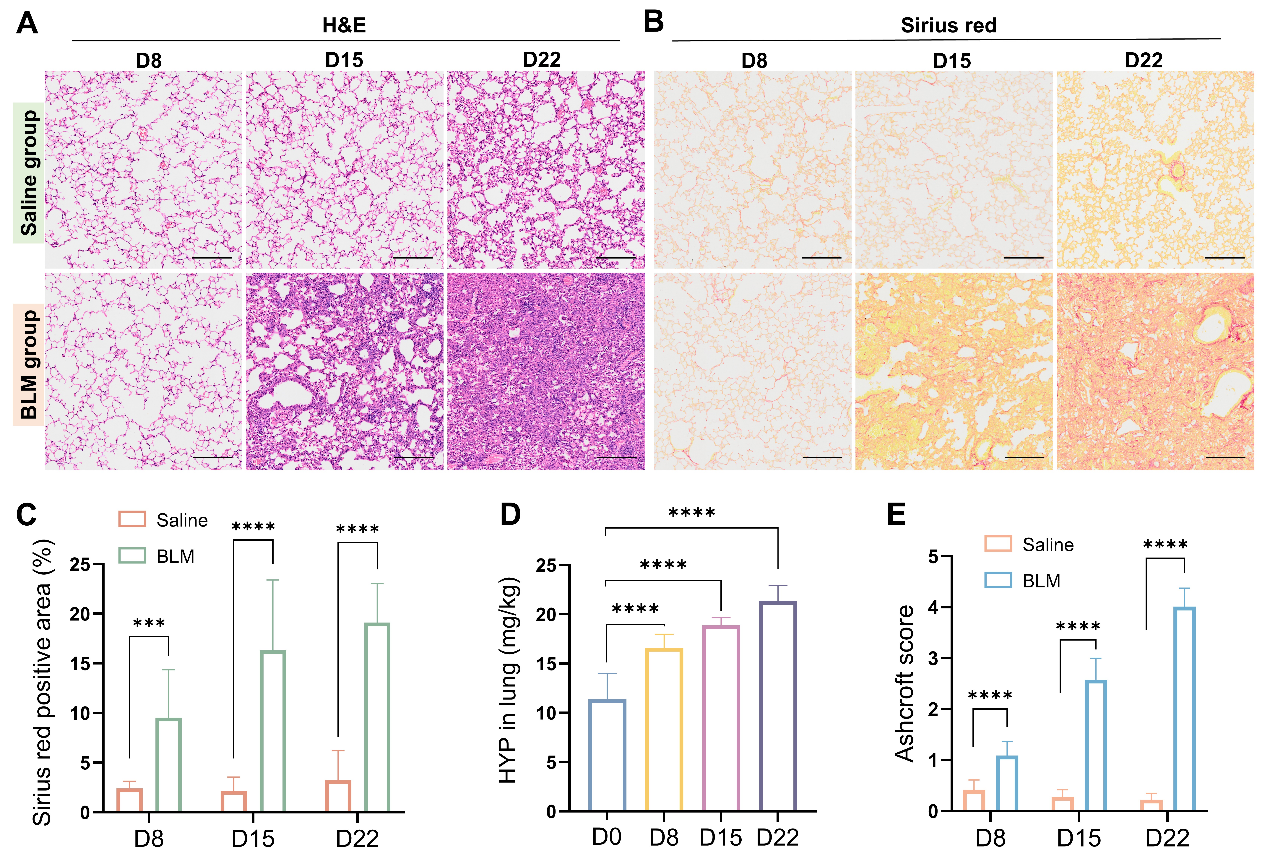


**Figure S5.** Identification of BLM-injured mice model. (A, B) Representative H&E and Sirius red stained images of lung in BLM and saline mice with longitudinal time points (D8, D15, D22; scale bar, 200 μm). (C) Positive area of Sirius red stained images from BLM and normal lung tissues. Red collagen was quantified using ImageJ software. (D) The HYP content in lung tissues. (E) Ashcroft score. ****p* < 0.001, *****p* < 0.0001.


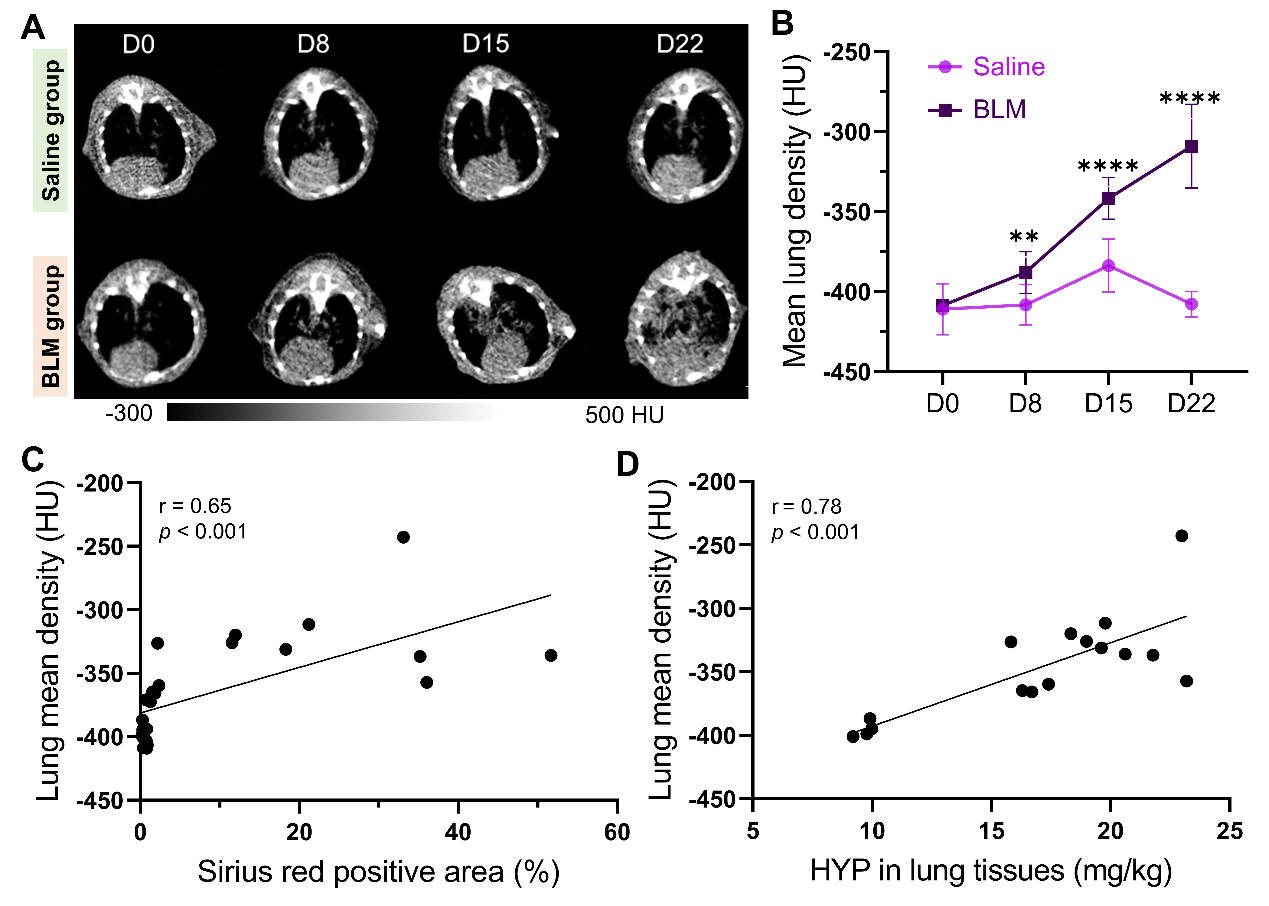


**Figure S6.** Longitudinal CT imaging of lung tissues in IPF and saline mice. (A,B) Representative CT horizontal images and comparison of mean lung density between two groups from D0 to D22 (n = 4). (C) Correlation between mean lung density and the positive area of Sirius red positive area. (D) Correlation between mean lung density and HYP content in lung tissues. ***p* < 0.01, *****p* < 0.0001.


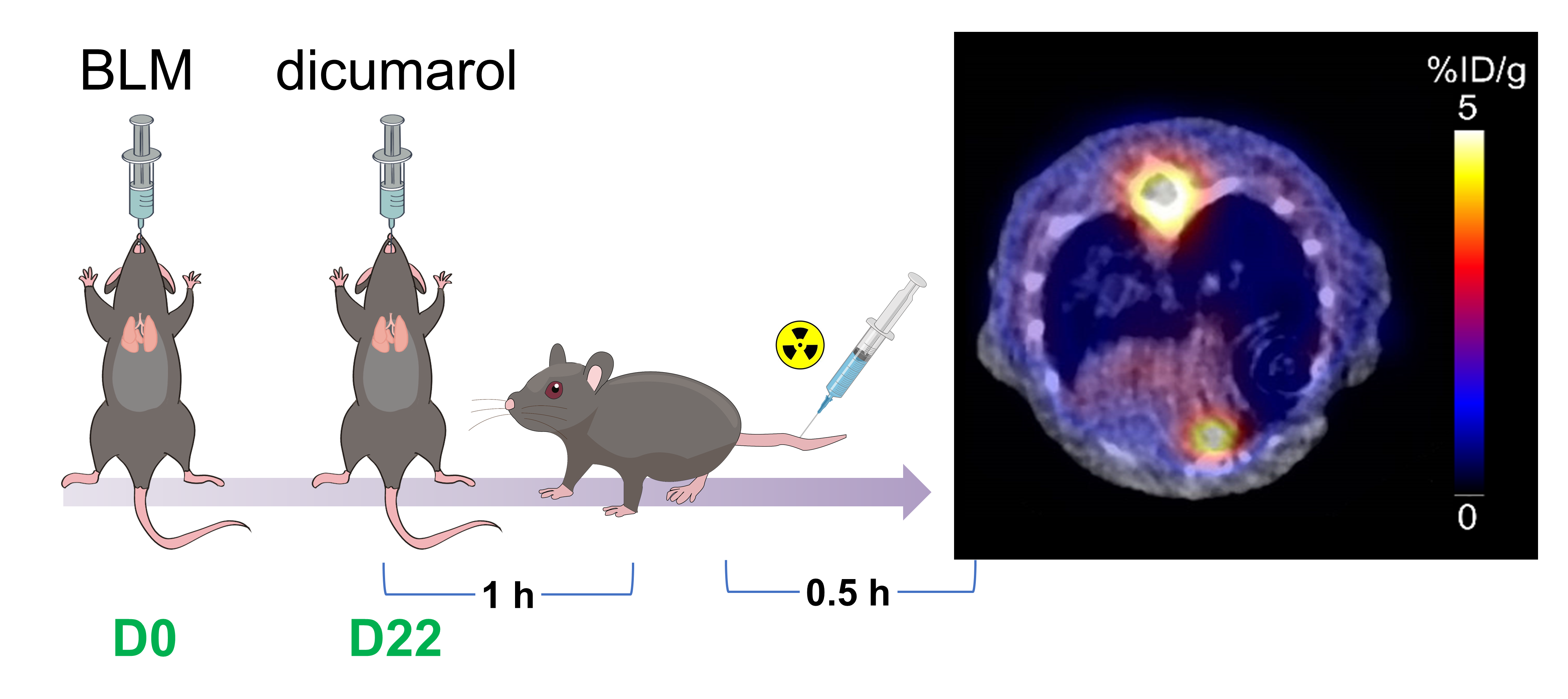


**Figure S7.** PET/CT imaging of ^18^F-NCRP in BLM-injured mice with NTR cleared.


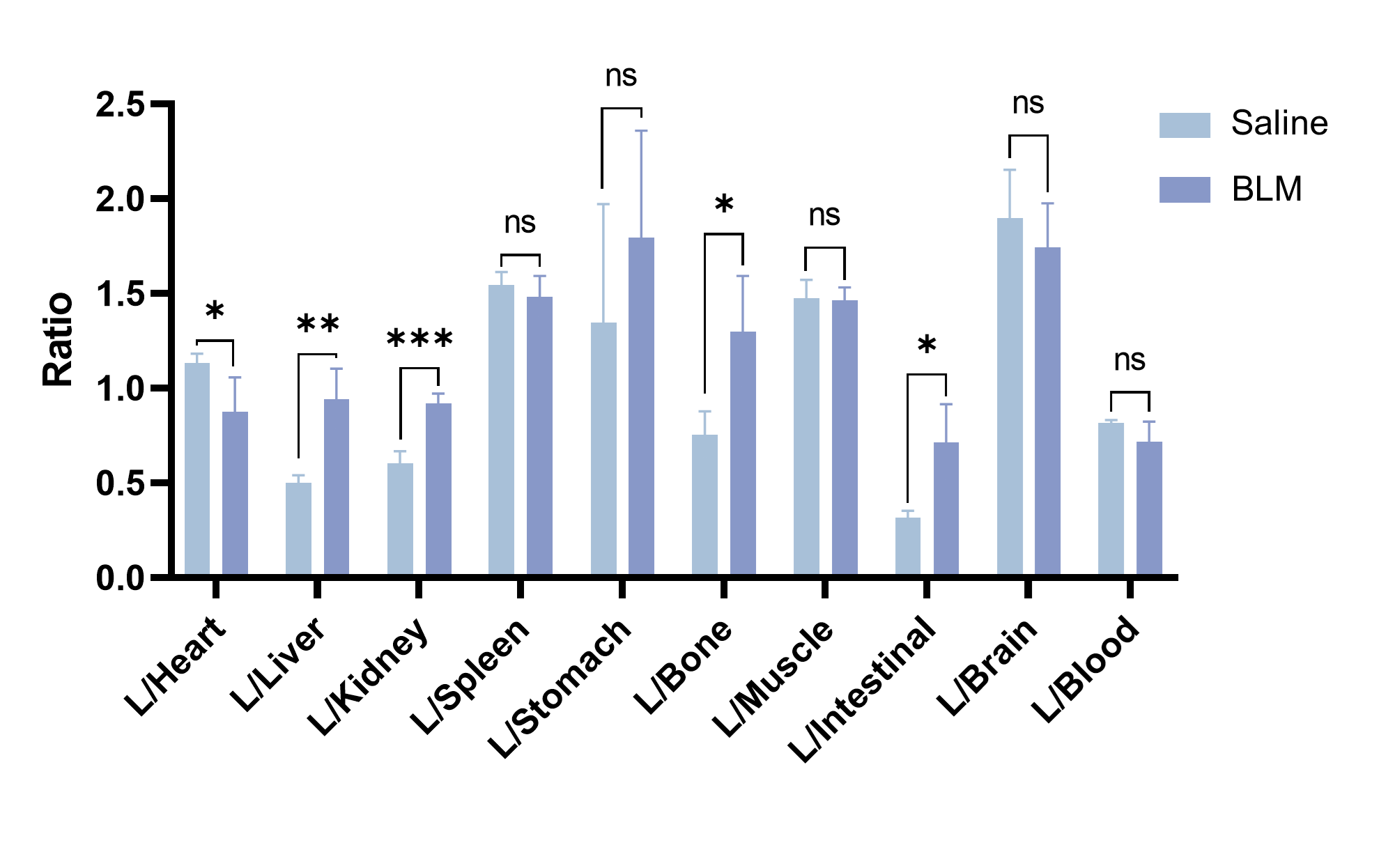


**Figure S8.** Comparison of lung-to-tissue ratios from biodistribution of BLM-injured and saline groups at 30 min on D22. L: lung. **p* < 0.05, ***p* < 0.01, ****p* < 0.001; ns, no significant difference.


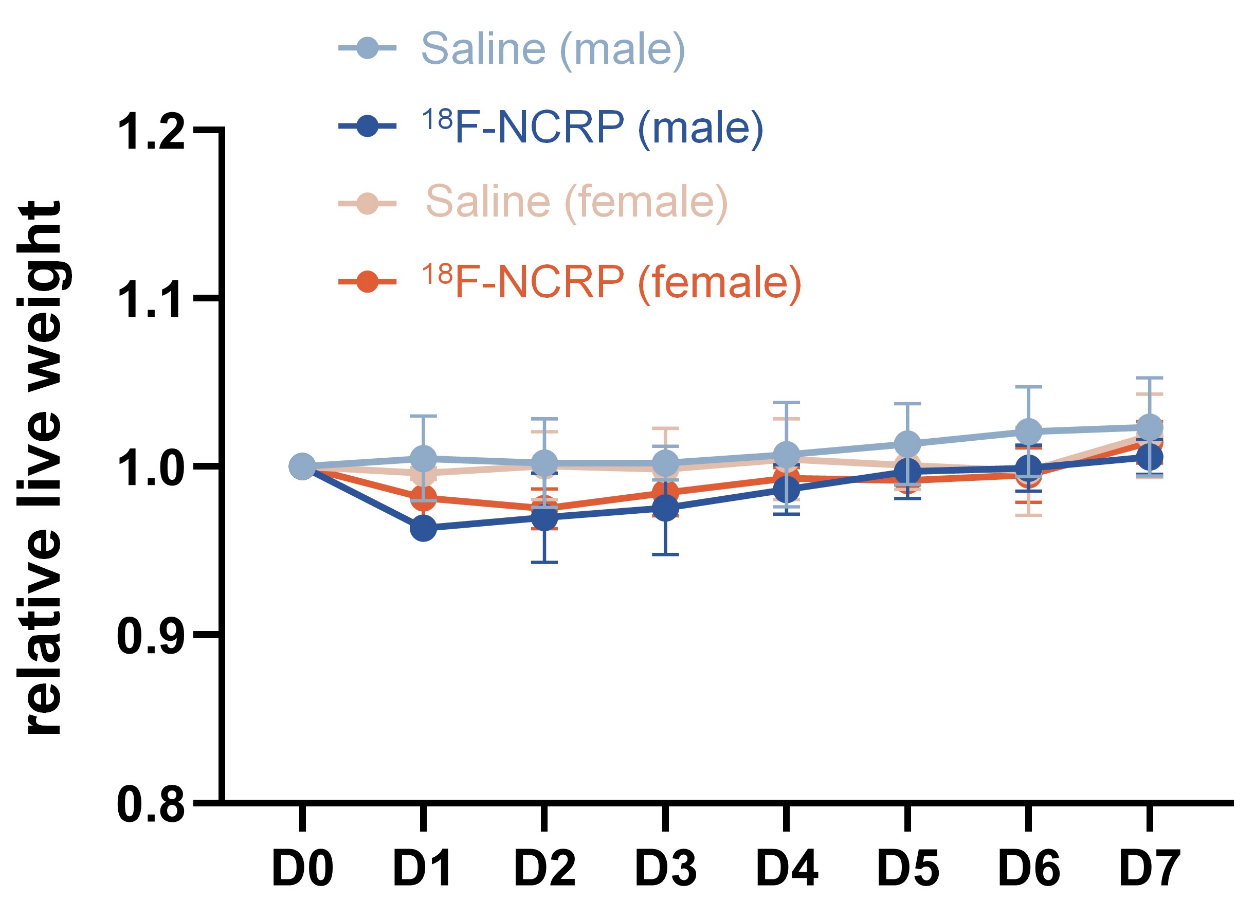


**Figure S9**. Weight change curve of mice after injection of 37 MBq of ^18^F-NCRP.


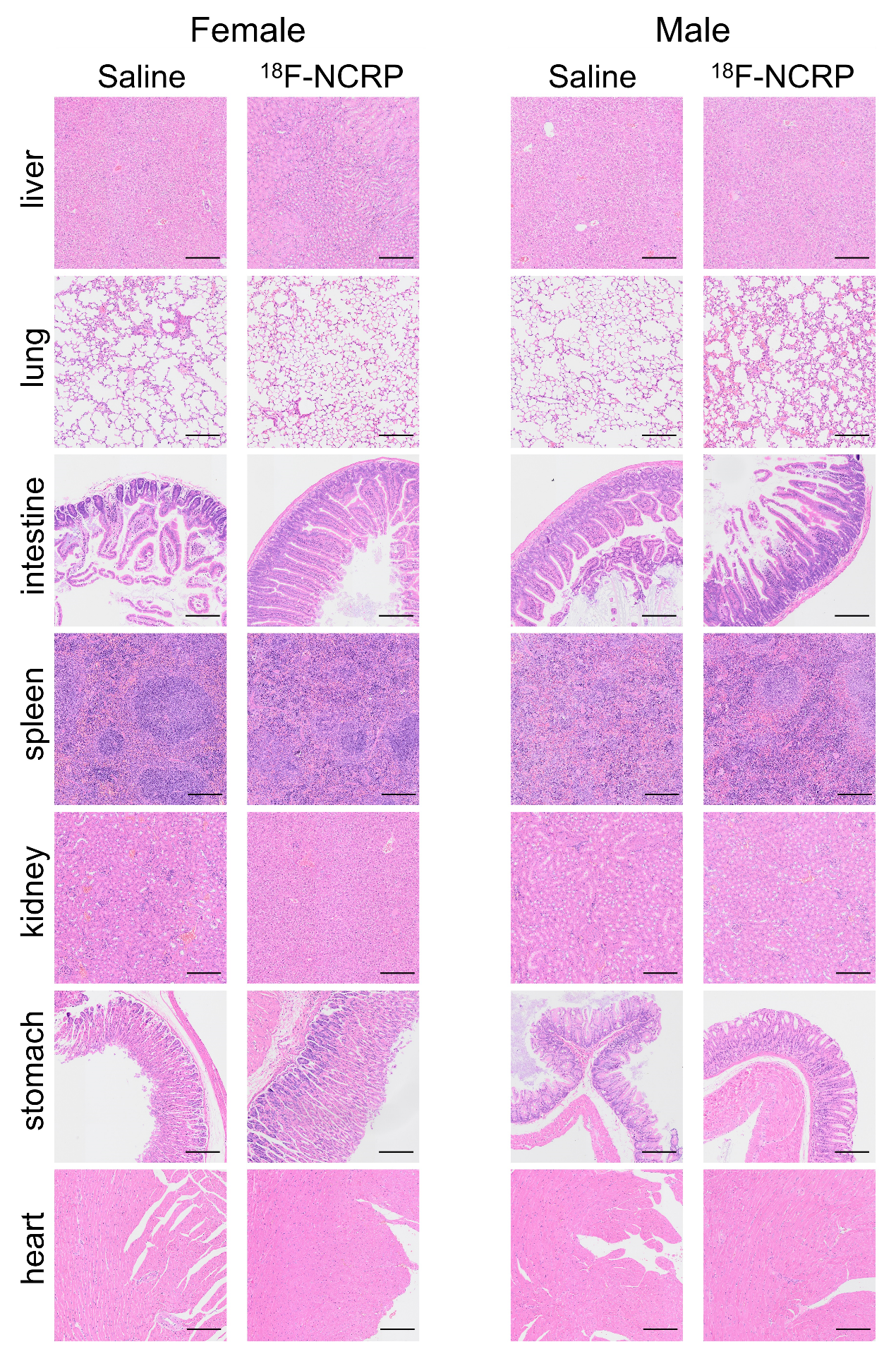


**Figure S10.** H&E staining of major organs in different group on the D7 after injection of ^18^F-NCRP (37 MBq). Scale bar: 200 μm.

**Table S1.** Biodistribution results of ^18^F-NCRP in BLM-injured and saline groups. Expressed as % injected dose per gram (%ID/g ± SD, n = 4).

|  | **BLM-injured group** | | | **Saline group** |
| --- | --- | --- | --- | --- |
|  | 30 min | 60 min | 90 min | 30 min |
| Heart | 7.67 ± 1.54 | 4.72 ± 0.31 | 4.42 ± 1.31 | 2.43 ± 0.15 |
| Liver | 7.07 ± 1.20 | 4.88 ± 0.57 | 3.71 ± 1.28 | 5.56 ± 0.38 |
| Lung | 6.55 ± 0.70 | 4.07 ± 0.46 | 3.56 ± 0.99 | 2.76 ± 0.17 |
| Kidney | 7.11 ± 0.37 | 4.34 ± 0.49 | 4.43 ± 0.17 | 4.60 ± 0.51 |
| Spleen | 4.43 ± 0.49 | 2.75 ± 0.44 | 3.83 ± 2.13 | 1.79 ± 0.16 |
| Stomach | 3.92 ± 1.39 | 3.43 ± 1.52 | 4.28 ± 1.54 | 2.36 ± 0.78 |
| Bone | 5.21 ± 1.22 | 5.92 ± 0.34 | 6.18 ± 1.36 | 3.70 ± 0.28 |
| Muscle | 4.47 ± 0.35 | 2.43 ± 1.82 | 3.26 ± 1.65 | 1.88 ± 0.14 |
| Intestine | 9.76 ± 3.12 | 3.98 ± 1.52 | 4.97 ± 1.16 | 8.86 ± 1.04 |
| Brain | 3.77 ± 0.30 | 2.66 ± 0.41 | 2.31 ± 0.88 | 1.48 ± 0.19 |
| Blood | 9.21 ± 1.24 | 9.09 ± 3.67 | 4.50 ± 1.52 | 3.39 ± 0.22 |

**Table S2**. ALT activity of mice serum was detected on the D7 after injection 37 MBq of ^18^F-NCRP. The normal reference range of ALT in mice is 10.06 – 96.47 U/L.

| Groups | Number | ALT (U/L) | Mean ± SD (U/L) |
| --- | --- | --- | --- |
| Saline (male) | M1 | 51.389 | 50.433 ± 0.860 |
|  | M2 | 49.303 |  |
|  | M3 | 50.606 |  |
| ^18^F-NCRP (male) | M1 | 57.006 | 54.788 ± 1.590 |
|  | M2 | 53.359 |  |
|  | M3 | 53.999 |  |
| Saline (female) | M1 | 46.426 | 46.717 ± 0.232 |
|  | M2 | 46.730 |  |
|  | M3 | 46.995 |  |
| ^18^F-NCRP (female) | M1 | 48.969 | 46.166 ± 2.141 |
|  | M2 | 43.774 |  |
|  | M3 | 45.754 |  |

**Table S3**. AST activity of mice serum was detected on the D7 after injection 37 MBq of ^18^F-NCRP. The normal reference range of AST in mice is 36.31 – 235.48 U/L.

| Groups | Number | AST (U/L) | Mean ± SD (U/L) |
| --- | --- | --- | --- |
| Saline (male) | M1 | 71.915 | 71.201 ± 0.591 |
|  | M2 | 70.467 |  |
|  | M3 | 71.221 |  |
| ^18^F-NCRP (male) | M1 | 87.924 | 88.935 ± 0.778 |
|  | M2 | 89.817 |  |
|  | M3 | 89.064 |  |
| Saline (female) | M1 | 84.621 | 85.254 ± 0.673 |
|  | M2 | 84.955 |  |
|  | M3 | 86.185 |  |
| ^18^F-NCRP (female) | M1 | 83.558 | 85.188 ± 1.157 |
|  | M2 | 85.874 |  |
|  | M3 | 86.132 |  |

**Table S4**. BUN activity of mice serum was detected on the D7 after injection 37 MBq of ^18^F-NCRP. The normal reference range of BUN in mice is 10.81 – 34.74 mg/dL.

| Groups | Number | BUN (mg/dL) | Mean ± SD (mg/dL) |
| --- | --- | --- | --- |
| Saline (male) | M1 | 30.6656 | 30.120 ± 0.417 |
|  | M2 | 30.0412 |  |
|  | M3 | 29.652 |  |
| ^18^F-NCRP (male) | M1 | 27.8488 | 27.335 ± 0.411 |
|  | M2 | 27.3112 |  |
|  | M3 | 26.8436 |  |
| Saline (female) | M1 | 25.715 | 25.855 ± 0.186 |
|  | M2 | 25.732 |  |
|  | M3 | 26.118 |  |
| ^18^F-NCRP (female) | M1 | 29.366 | 27.423 ± 1.969 |
|  | M2 | 24.724 |  |
|  | M3 | 28.179 |  |

**Table S5**. CR activity of mice serum was detected on the D7 after injection 37 MBq of ^18^F-NCRP. The normal reference range of CR in mice is 10.91 – 85.09 μmol/L.

| Groups | Number | CR (μmol/L) | Mean ± SD (μmol/L) |
| --- | --- | --- | --- |
| Saline (male) | M1 | 22.78 | 22.628 ± 0.505 |
|  | M2 | 21.948 |  |
|  | M3 | 23.157 |  |
| ^18^F-NCRP (male) | M1 | 21.326 | 22.061 ± 0.521 |
|  | M2 | 22.392 |  |
|  | M3 | 22.465 |  |
| Saline (female) | M1 | 18.82 | 19.745 ± 0.917 |
|  | M2 | 20.995 |  |
|  | M3 | 19.419 |  |
| ^18^F-NCRP (female) | M1 | 17.753 | 21.075 ± 2.692 |
|  | M2 | 21.126 |  |
|  | M3 | 24.346 |  |
